# Anthocyanins in Whole Grain Cereals and Their Potential Effect on Health

**DOI:** 10.3390/nu12102922

**Published:** 2020-09-24

**Authors:** Alyssa Francavilla, Iris J. Joye

**Affiliations:** Department of Food Science, University of Guelph, Guelph, ON N1G2W1, Canada; ijoye@uoguelph.ca

**Keywords:** anthocyanins, antioxidants, whole grains, functional cereals, health benefits

## Abstract

Coloured (black, purple, blue, red, etc.) cereal grains, rich in anthocyanins, have recently gained a lot of attention in the food industry. Anthocyanins are water-soluble flavonoids, and are responsible for red, violet, and blue colours in fruits, vegetables, and grains. Anthocyanins have demonstrated antioxidant potential in both in vitro and in vivo studies, and the consumption of foods high in anthocyanins has been linked to lower risks of chronic diseases. As such, whole grain functional foods made with coloured grains are promising new products. This paper will review the characteristics of cereal anthocyanins, and assess their prevalence in various commercially relevant crops including wheat, barley, maize, and rice. A brief overview of the antioxidant potential, and current research on the health effects of cereal-based anthocyanins will be provided. Finally, processing of coloured cereals in whole grain products will be briefly discussed. A full understanding of the fate of anthocyanins in whole grain products, and more research targeted towards health outcomes of anthocyanin supplementation to/inclusion in cereal food products are the next logical steps in this research field.

## 1. Introduction

There is a large body of evidence to suggest that the inclusion of whole grains in the diet is beneficial for human health [1,2]. Whole grain cereals, i.e., grains that include the bran, germ, and endosperm, are good sources of vitamins, minerals, dietary fibre, and phytochemicals [1,2]. These compounds are mostly present in the outer kernel layers, i.e., the pericarp and aleurone, which are typically removed when the grain is milled and cereal flour is produced [1].

The intake of high levels of dietary fibre, vitamins and minerals, and important phytochemicals like indoles, polyphenols, carotenoids, and phytosterols is considered to be responsible for the potential health-promoting effect of regular cereal whole grain consumption [1,2]. The relationship between the regular consumption of wholemeal products and a reduced risk of non-communicable chronic diseases has been previously established [2,3]. The specific effects of food structure and dietary fibre content, combined with the antioxidant and anti-carcinogenic effects of numerous compounds (especially polyphenolic compounds), are well recognised actors in the risk reduction of cardiovascular diseases, obesity, diabetes, and certain types of cancer [1,3].

Most research has focused on the health effects of the major constituents of whole grains (e.g., dietary fibre) [1]. However, bioactive small molecules also play a role in the benefits of whole grain consumption. Polyphenols, for example, are compounds that possess one or more aromatic rings, with one or more hydroxyl groups, and can be categorised into one of five groups (flavonoids, stilbenes, phenolic acids, coumarins, and tannins) [1]. Phenolics act as singlet oxygen quenchers and free radical hydrogen donors, which have a protective effect on cell constituents against oxidative damage [4]. Among them, anthocyanins are a group of water-soluble flavonoids and are responsible for red, violet, and blue colours in fruits, vegetables, and cereal grains [5,6]. Anthocyanins have demonstrated in vitro antioxidant potential, and consumption of foods high in anthocyanins has been linked to a lower risk of chronic diseases [5].

Several commercially relevant cereal grain families contain some distinctly coloured varieties [7]. These colours often stem from anthocyanins present in the kernels as one of the primary natural pigments. Wheat, maize, sorghum, barley, rice, and millet all have varieties with significant amounts of anthocyanins in the outer layers of the kernel. Coloured grains rich in anthocyanins have been identified as promising ingredients for the development of whole grain functional foods. This paper will review the structure and function of anthocyanins and the antioxidant potential of cereal anthocyanins. Additionally, the prevalence of anthocyanins in various whole grains will be discussed in detail.

## 2. Structure and Function of Anthocyanins

**Figure 1:**
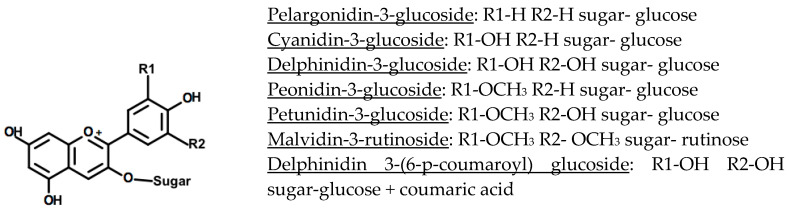
The structures of six common anthocyanins and one acylated anthocyanin, with the individual ring substituents listed below the flavylium ion backbone [9,16].

Anthocyanins are water-soluble phenolic pigments responsible for red, purple, blue, or even black colours in fruits, vegetables, grains, flowers, and other pigmented plant tissues [5,6,8,9]. All anthocyanins share the same core structure, a flavylium ion, consisting of two aromatic ring structures linked by a three-carbon heterocyclic ring that contains oxygen (Figure 1) [6,8,10]. The anthocyanidin (aglycone form) is the core structure of the anthocyanin. The addition of a sugar side chain results in the glycosidic form of the anthocyanidin molecule, called an anthocyanin [6,10]. Anthocyanidins, as well as their glycosylated and acyl glycosylated forms, can be found in nature [6,11]. Over 23 anthocyanidins and 500 different anthocyanins have been isolated from plants [7,12,13]. Anthocyanidins differ in their hydroxylation and methoxylation degree and pattern. The large diversity in anthocyanins does not only stem from the variability in the anthocyanidin core structure, they also differ in the nature and number of sugars attached to the core structure, as well as the nature and number of side chains attached to these sugar residues [6,9,14]. The most common sugar residues found in anthocyanins are glucose, xylose, rhamnose, arabinose, and galactose [6,9,14]. While monosaccharide functional groups are more common, di- and tri-saccharide groups are also found [14]. Sugars are most commonly linked to the aglycone core at position 3 (Figure 1) [9,14]. When multiple sugar groups are present, these additional sugar moieties are often linked to positions 5 and/or 7 of the aglycone core structure (Figure 1) [6,9,14]. The acylglycosidic form occurs when sugar groups are acylated with aliphatic, hydroxybenzoic, or hydroxycinnamic acids [9,15]. The most commonly found acids are malonic, acetic, and caffeic acids [14].

Despite the wide structural variations present in anthocyanin structures, six main anthocyanidin compounds are commonly found in food products [6,11]. About 50% of anthocyanins found in fruits and cereals are cyanidin derivatives, followed by pelargonidin (12%), delphinidin (12%), peonidin (12%), petunidin (7%), and malvidin (7%) derivatives [11]. The conjugated bonds of the anthocyanidin moiety are responsible for the molecule’s colour [8,11]. Anthocyanidin compounds vary in the degree of ring hydroxylation and methoxylation [6,8,13]. The level of hydroxylation/methoxylation generally influences the hue of the compound as well as the molecule’s stability, with increased hydroxylation increasing the anthocyanin’s blueness and reducing its stability [9]. Increased methoxylation, on the other hand, increases the anthocyanin’s redness and molecular stability [6,8,9,10].

In addition to the chemical structure of anthocyanins, the anthocyanin concentration, temperature, and pH, as well as exposure to light, oxygen, enzymes, ions, other flavonoid and phenolic compounds, ascorbic acid, and sulphites affect the stability of the anthocyanin molecules [9,10,17]. The anthocyanidin backbone of the molecule is highly reactive due to the electron-deficient flavylium cation structure [6,10,17]. This (deglycosylated) molecular structure is particularly vulnerable to nucleophilic attack by water; however, the glycosylated and acylated anthocyanins can also suffer from this degradation [6,9,10]. Anthocyanins are often partially degraded by the combined action of cellular and environmental factors [10,17]. One of the degradation patterns shows a breakdown from the quinoidal base to the flavylium ion, followed by a conversion to the carbinol pseudobase and chalcone, which is heat labile and easily cleaved [9,15]. The rate of this degradation is controlled significantly by temperature, with higher temperatures resulting in decreased stability [9,15]. Co-pigmentation also plays a large role in the stability of cereal anthocyanins, but may also impact the absorption of compounds during digestion [6,9,10,18,19]. The electron-deficient flavylium ion associates with co-pigments that are rich in electrons, having a net stabilising effect by protecting the flavylium cation from nucleophilic attack by water at position 2, and from other species (e.g., ascorbic acid, peroxides) at position 4 (Figure 1) [15]. Co-pigmentation can occur through a variety of interactions and with a range of co-pigments including other anthocyanins, aglycones, metals, or other phenolic compounds [6]. Acylated anthocyanins are also more stable than alternate forms, due to the protection from nucleophilic attacks conferred by the acyl groups [10,16]. The stability conferred by the acyl group is dictated by the location, type, and number of acyl groups that have been esterified to the anthocyanin [16]. While any of the anthocyanidin hydroxyl side groups can be esterified by organic acids under specific conditions, most commonly, acylation occurs on the glycosyl –OH groups [16]. Acyl groups can stack around the flavylium ring and protect it from degradation, due to the flexibility of the associated sugar groups [15,16]. Acyl groups have been found to negatively affect the absorption of anthocyanins after digestion [19].

Anthocyanin analysis is predicated on efficient extraction and identification. Multiple methods have been developed to achieve this [6,9,10]. Most commonly, crude solvent extraction methods are used on ground whole grains. Polar organic solvents (methanol, ethanol, or acetone) are combined with an acid (hydrochloric acid, formic acid, etc.) to create an acidic solvent [6,14]. Methanol and hydrochloric acid are often used due to their efficacy and minimal hazard level, and due to their status as common lab materials [14]. Acidification of the solvent allows for the anthocyanins to be extracted in a stable form, although it can result in partial hydrolysis of acyl moieties present [6,14]. This can be minimised with the use of weak acids [6,14]. Recent advances include the use of pressurised liquid extraction, supercritical fluid extraction, accelerated solvent extraction, and microwave-assisted solvent extraction [6,14,20]. Several anthocyanin quantification techniques exist that differ largely in their complexity. The spectrophotometric method first published by Hucl [21], wherein the anthocyanin concentration in an extract is measured at the wavelength of maximum cyanidin absorbance (i.e., 520–525 nm) and is corrected for molar absorptivity, is most commonly used. This simple method provides the total anthocyanin content (TAC) expressed as cyanidin-3-glucoside equivalents per weight of the starting material [21]. The differential pH method is also utilised for anthocyanin determination, and is predicated on the anthocyanin’s structural and absorption changes at different pH values, where the compound is red at pH 1.0, and turns colourless at pH 4.5 [22]. Alternatively, the TAC can also be derived from chromatographic methods used for identifying the different anthocyan (id) in species. High-performance liquid chromatography (HPLC), for example, is used with various detectors for this purpose. There is a strong correlation between the measured TAC results obtained with the spectrophotometric method and the HPLC-UV/Vis method [23]. UV/Vis detectors can be used for general anthocyanin determination in HPLC analysis, allowing for the identification (based on retention times) and quantification of the main anthocyanins and anthocyanidins (cyanidin, pelargonidin, etc.) but can present some difficulty when discriminating between different side chains [7,14]. The use of more specific detectors such as LC/MS (Liquid Chromatography-Mass Spectroscopy), and PDA-MS (Photo Diode Array-Mass Spectroscopy) can result in a more detailed understanding of the presence of side chains, including sugar moieties and acidic residues [6,14].

Anthocyanin analysis also includes a variety of assays that focus on the antioxidant potential of cereal anthocyanins. However, when utilising these methods, it can be difficult to distinguish the effect of the anthocyanins from that of other antioxidants (e.g., phenolic acids, coumarins, etc.) present in whole grains. Testing isolated extracts is one strategy to distinguish between anthocyanins and other phenolics. Some commonly utilised assays include DPPH-free radical assay, oxygen radical antioxidant capacity (ORAC), Trolox equivalent antioxidant capacity (TEAC), and ferric reducing antioxidant power (FRAP) analyses [24]. These are used alone, or in combination, to determine the antioxidant potential of the cereal grain in general, or the cereal anthocyanins in particular [24]. Differences in antioxidant potentials suggest that the reducing, radical scavenging, and iron chelating capacities of phenolic and flavonoid compounds vary depending on their structure [25].

## 3. Anthocyanins in Whole Grain Cereals

### 3.1. Wheat

Wheat (*Triticum aestivum*) is consumed by humans in large quantities globally, and is second only to rice as a cereal component in the human diet [7,26,27]. The non-anthocyanin-containing white and red wheat varieties are most commonly consumed, while blue, purple, and black wheat varieties are either not cultivated on a large scale or are predominantly grown as a speciality crop. Although wheat species have been studied to gain insight on the anthocyanin content, structure, and profile, the exact anthocyanin composition of these grains is still largely unknown [28]. This high level of uncertainty on coloured wheat anthocyanin structure and profile (i.e., the relative occurrence of each anthocyanin) is mainly due to large differences observed between wheat varieties and differences in extraction methods. Novel identification methods such as HPLC-MS have made strides in clarifying the presence and levels of the different anthocyanins in plants. These methods have even shed light on the prevalence of less common anthocyanins in plants.

**Figure 2:**
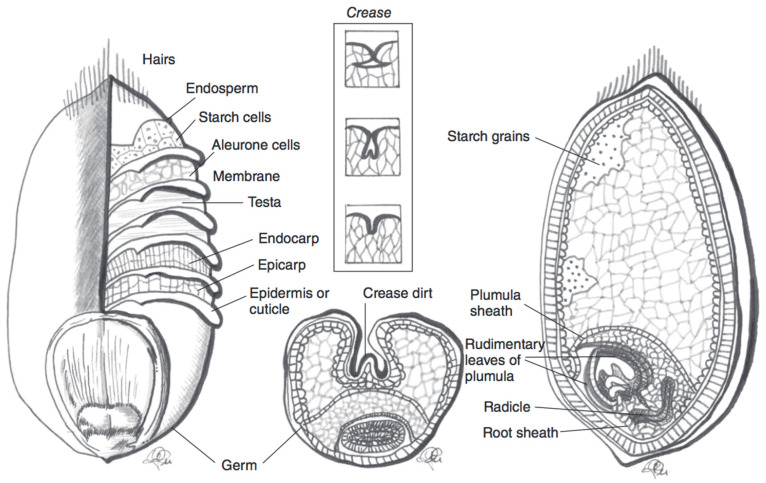
Schematic representation of a wheat kernel cross-section, with component parts. The pericarp is represented by the endocarp and esocarp in this illustration. Adapted from reference [27].

Blue, purple, and black wheat species contain large quantities of anthocyanins in the outer kernel layers (Figure 2). In purple wheat, anthocyanins are localised in the pericarp, whereas in blue wheat they are found in the aleurone layer. In black (also referred to as “deep purple”) wheat, anthocyanins can be found in both the pericarp and the aleurone layer [29]. The distribution of anthocyanins in the outer kernel layers affects the extractability and stability of these compounds in the wheat grain. Purple wheat has, on average, a lower TAC than blue and black wheat varieties. It is theorised that, in blue and black varieties, the pigment location leads to an enhanced anthocyanin stability [30,31]. The superior colour stability stems from the protection that several layers of pericarp confer to the underlying aleurone layer [30]. Indeed, it has been shown that the pericarp of wheat kernels is often damaged during harvest or subsequent transport [32]. It has also been observed that anthocyanin pigments are more tightly bound in the pericarp than in the aleurone layer, therefore making them relatively less extractable from purple wheat varieties. Anthocyanin content and localisation can be determined from the genetic profile of the wheat [30]. The challenge of developing commercially relevant coloured wheat varieties can be mainly brought back to issues of limited harvest yield [30]. Blue aleurone genes have been linked to negative impacts on yield [30]. Finding a strategy to disrupt the link between anthocyanin production and yield would be beneficial for wheat breeders to develop a higher yield coloured wheat variety [30].

The anthocyanin compounds present in coloured wheat vary based on wheat variety, as well as growing conditions [33]. The influence of growing conditions and kernel maturity makes definitive anthocyanin identification and quantification challenging, even within one variety [33]. The bioaccumulation of phenolic compounds is not yet well understood [34,35]. Growth temperature and genotype have been found to correlate with total phenolic compounds [35]. Rain levels have also been demonstrated to affect TAC in grains [36]. Moreover, while identification methods have improved in sensitivity and specificity, there are still unidentified anthocyanins. Many studies have been performed to elucidate the identity and quantity of wheat anthocyanins. An overview of data, from a selection of studies, is presented in Table 1.

The anthocyanin profile of black, blue, and purple wheat is complex and diverges between varieties. In Laval and Konini purple wheat varieties, Abdel-aal [28] found that the most abundant anthocyanin is cyanidin-3-glucoside (4 mg/g), followed by peonidin-3-glucoside (2 mg/g), cyanidin-malonyl-glucose (1 mg/g), and cyanidin-succinyl-glucose (1 mg/g), along with peonidin-malonyl-glucose, peonidin-succinyl-glucose, and peonidin-malonyl-succinyl-glucose. Purple wheat anthocyanins are more often acylated than the anthocyanins found in blue and black wheat [30]. It is unclear whether this observation is due to the location of anthocyanins (pericarp vs. aleurone), or to other unknown factors. The TAC of purple wheat has been found to range between 10 and 305 mg/kg dry kernel weight [24,28,31]. In one blue wheat variety, delphinidin-3-glucoside is the most prominent anthocyanin (57 mg/g), followed by delphinidin-3-rutinoside (41 mg/g), cyanidin-3-glucoside (20 mg/g), and cyanidin-3-rutinoside (17 mg/g), along with petunidin-3-glucoside, petunidin-3-rutinoside, and malvidin-rutinoside [28]. The TAC of blue wheat ranges between 17 and 211 mg/kg dry kernel weight [5,28,30,31,37,38]. Finally, black wheat has been less well characterised, possibly due to the limited availability of black wheat varieties. The black wheat TAC is thought to vary between 56 and 198 mg/kg dry wheat kernel, and an average of 120 ± 10 mg/kg dry kernel weight can be calculated [30,31]. Twenty-six distinct anthocyanin compounds were identified in a black wheat variety and two of its ancestral lines [30]. All six major anthocyanidins were represented (i.e., cyanidin, delphinidin, pelargonidin, petunidin, malvidin, and peonidin) with glucoside and rutinoside linkages [30]. Additionally, some anthocyanins had tri-saccharide linkages, such as rutinoside associated with a pentose sugar [30].

### 3.2. Barley

**Figure 3:**
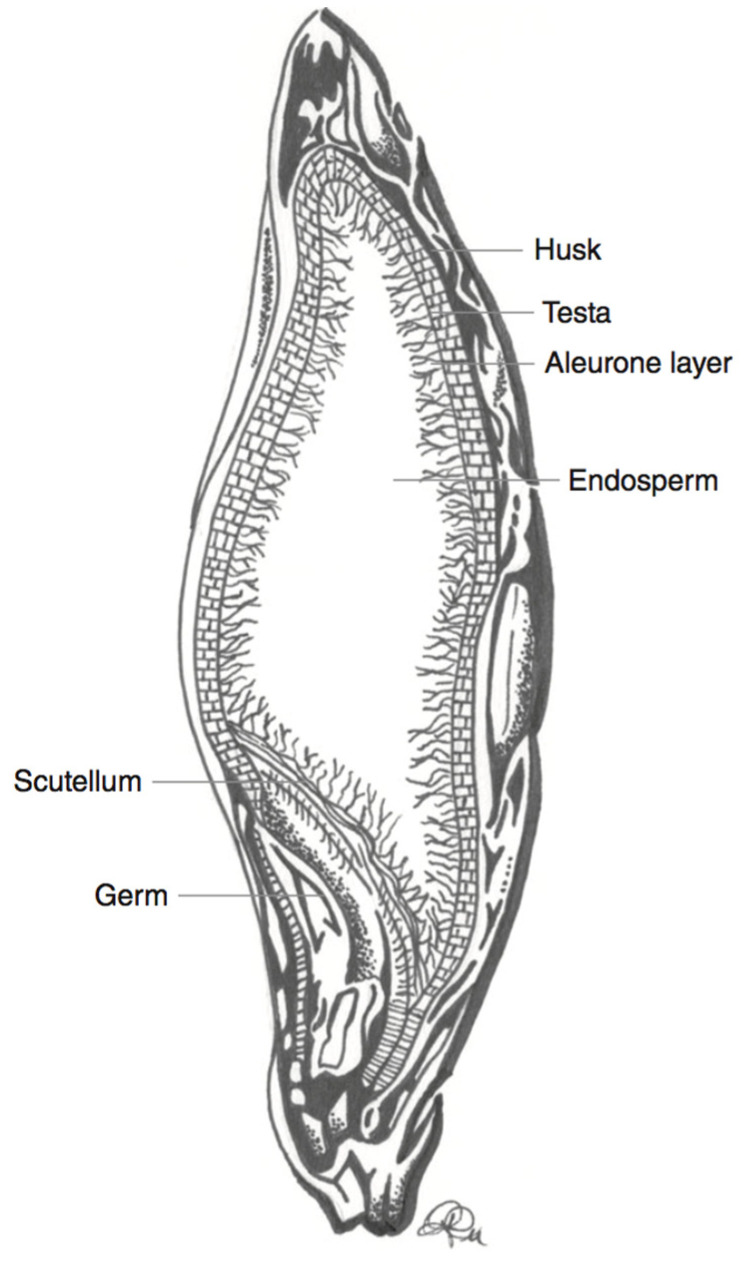
Schematic representation of a barley kernel cross-section, with component parts. Adapted from reference [27].

Barley (*Hordeum vulgare*) is another major cereal grain that is grown in temperate climates [27]. Barley is often used in fermentation processes as a sugar source. An example of this is during the production of alcoholic beverages [27]. Barley also serves as animal feed, and can be used as an edible grain (cooked berries). Barley cultivars are usually yellow to amber in colour, but the seeds can also take on more uncommon colours such as white, purple, blue, and black [27]. Both hulled and hull-less varieties of barley exist [27,39]. The fibrous hull is inedible, and is typically removed before consumption [27]. Dehulled (and hull-less) barley grains can then be further processed for human consumption. Barley seeds may be left as whole berries (pot barley), or the bran may be removed in a pearling process [39]. Both of these products can be further milled into a fine flour [39]. However, as is the case for other cereals, the anthocyanins in barley are localised in the outer kernel layers, and more specifically in the pericarp or aleurone cell layers for purple and blue barley, respectively (Figure 3) [38,40]. Therefore, similar to what is found for wheat, milling usually results in a fine flour that does not contain significant anthocyanin levels (after the removal of the bran layers). Purple and blue barley cultivars have a higher average anthocyanin content than their black counterparts [38]. This is likely due to the contribution of a melanin-based pigment to black barley pigmentation, rather than sole reliance on anthocyanins for colour [38].

As with wheat, growing conditions and genotype influence the type and quantity of anthocyanins in the grain (Table 2) [33].

The anthocyanin profile and quantity of barley vary between studies. In black barley, the TAC varied from 0 to 77 ± 17 mg/kg of kernel [38,39,40]. The anthocyanin types that were found in the black barley samples were dissimilar between varieties. Using LC-MS, peonidin derivatives were identified as the main anthocyanins [40]. Conversely, using HPLC-UV/Vis, delphinidin-3-glucoside was identified as the main anthocyanin in another purple variety, followed by peonidin-3-glucoside and malvidin-3-glucoside [39]. In blue barley, using LC-MS—similarly to black cultivars—peonidin derivatives were identified as the dominant anthocyanins [40]. In another study, LC-MS resolved cyanidin-3-glucoside and petunidin-3-glucoside, which were found to be the most prevalent anthocyanins [41]. Conversely, using HPLC, petunidin-based anthocyanins were not detected, while cyanidin-3-glucoside and delphinidin-3-glucoside were [41,42]. Blue barley has a TAC between 35 and 84 mg/kg dry kernel weight, while purple barley has anthocyanin contents between 573 and 679 mg/kg dry kernel weight [28,38,39,40,41,42]. In purple barley, a more complex anthocyanin profile was found. Using LC-MS, cyanidin-3-glucoside, pelargonidin-3-glucoside, peonidin-3-glucoside, cyanidin-3-(6″ succinyl) glucoside, and peonidin-3-(6″ succinyl) glucoside, as well as various other cyanidin and peonidin derivatives, are found [39,42]. HPLC-UV/Vis analysis confirmed this anthocyanin profile, identifying peaks for cyanidin, pelargonidin, and peonidin-3-glucoside [42]. Hulled barley cultivars had lower anthocyanin contents than their hull-less counterparts, across all three coloured varieties [42]. However, the hull fraction was found to also contain anthocyanins [42]. As such, a portion of the total anthocyanins would be lost upon processing/dehulling [42].

### 3.3. Maize

**Figure 4:**
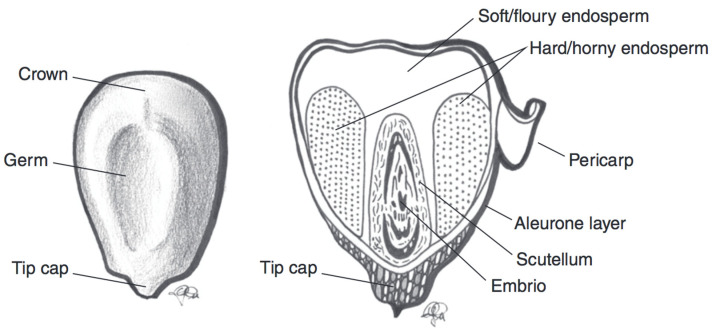
Schematic representation of a maize kernel cross-section, with component parts. Adapted from reference [27].

Maize (*Zea mays*) is widely consumed in Mexico and Central America [27]. The major use of maize in the human diet, however, involves the isolation of corn starch and its use as a refined food ingredient [27,43]. The classification of maize into subspecies is primarily based on the amount and type of starch in the kernel [27,43]. The maize subspecies include dent, waxy, and sweet corn species, among others. There also exists great genetic diversity in maize kernel colour [43,44]. Black, blue, pink, red, purple, and brown maize varieties exist [43]. In purple maize kernels, as with wheat and barley, anthocyanins are found in the pericarp, while in blue varieties, anthocyanins are found in the aleurone layer. In black and dark red kernels, anthocyanins are found in both the aleurone and pericarp layers (Figure 4) [44]. A selection of the current data for TAC in various coloured maize species is outlined in Table 3.

Variations in anthocyanin profile can be observed between different maize colours, as well as between waxy and other maize subspecies. When testing ornamental maize varieties using LC/MS, a very complex anthocyanin profile was resolved [28]. Upon in-depth analysis of the anthocyanin profile in Shaman Blue, Cutie Pink, purple, Scarlet Red, and Fiesta maize varieties, a minimum of 15 distinct anthocyanins were identified (Fiesta) [28]. The Scarlet Red maize variety even had 27 distinct anthocyanin peaks [28]. All of the tested varieties had cyanidin-3-glucoside as the main anthocyanin [28]. Most of the anthocyanins in the coloured maize varieties were glycosylated and some were acylated (purple and scarlet) [28]. However, a substantial amount of aglycones (cyanidin, peonidin, pelargonidin) was also found [28]. The aglycones found could have been present in the corn prior to extraction and quantification, or were produced due to anthocyanin degradation during processing. In the waxy corn varieties tested, the anthocyanin profile is somewhat less intricate, with only ~7 to 10 anthocyanin compounds identified [45]. In all varieties, cyanidin derivatives were predominant [45]. Acylated cyanidins, including cyanidin-3-(6″ malonyl glucoside) and cyanidin-3-(3″,6″-dimalonyl glucoside), were the majority of these, making up between 46–84% of the total anthocyanins in waxy corn [45]. In general, coloured maize varieties were found to contain between 27 to 1439 mg anthocyanins/kg of dry kernel [28,44,45,46,47]. When classified according to the different coloured varieties, the average TAC (in mg/kg dry kernel weight) was between 99 to 379 (blue), 26.5 to 1439 (purple), 76.2 to 120 (black), and 2.5 to 696 (pink/red) [28,44,45,46,47]. The differences in TAC reported in these studies may be due to various factors, including differences between genotypes, developmental stages, growing conditions, and quantification and extraction methods [33]. Milk stage maize kernels were consistently shown to have lower TAC than mature kernels, indicating that anthocyanins are formed or deposited in the kernel during maturation [48]. Therefore, slight differences in maturation level at harvest can have a large impact on the anthocyanin content [48].

### 3.4. Rice

**Figure 5:**
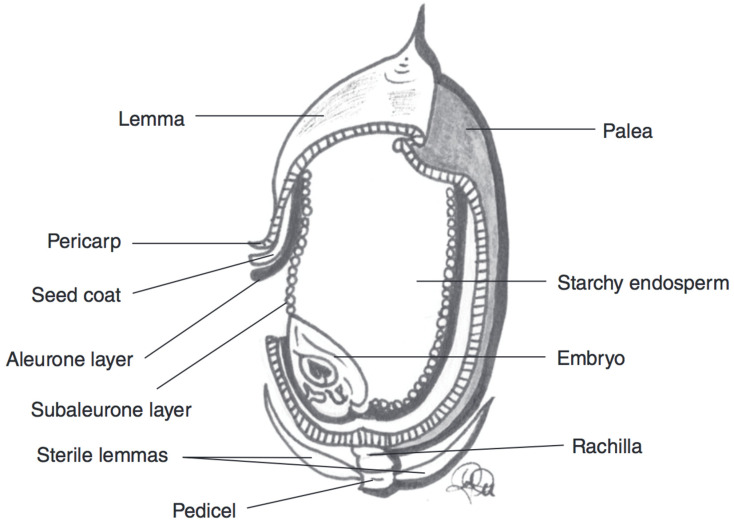
Schematic representation of a rice kernel cross-section, with component parts. Adapted from reference [27].

Rice is the most consumed cereal grain in Asia [27]. Rice is either consumed in a polished form (the bran layer is removed), as a milled flour, or eaten as a whole grain (with the bran layer intact). Colloquially, rice with its bran layer intact is referred to as brown rice [4,27]. However, the terminology “brown” refers to the presence of the darker coloured bran rather than the hue of the kernel [4,27]. The USDA National Small Grains Collection classifies rice into seven different colour groups: purple, variable purple, red, brown, speckled brown, light brown, and white coloured rice [49]. The most widely cultivated and consumed rice is light brown in colour [4,27]. However, the coloured rice varieties classified as red and purple have been the focus of increased interest due to potential health benefits. In coloured rice kernels, anthocyanins are also believed to accumulate in the outer layers of the kernels. However, studying the exact localisation of anthocyanins in rice kernels is hampered by the difficulty of separating the outer layers of a rice kernel [49]. After polishing, Shao [50] found that only 3% of TAC was retained in the kernels relative to the whole kernels. It is hypothesised that anthocyanins are localised similarly in rice kernels to other cereal grains; with anthocyanins in the pericarp of purple varieties, the aleurone of blue varieties, and with anthocyanins in both locations of black varieties (Figure 5). A selection of some reports on the TAC in rice is displayed in Table 4.

In agreement with the other grains, rice varieties display a large variability in their anthocyanin profiles and contents, with kernel colour and type (glutinous vs. non-glutinous) playing the largest role. Additionally, the method used to analyse the profile contributes to the specificity of the results. Again, growing and harvesting conditions impact the TAC substantially [33]. Over a two-year period, the same variety of rice was grown, harvested, and tested twice, and the results showed variance in the contents of individual and total anthocyanins [49]. Overall, anthocyanin content decreased in the second year, with disproportionate decreases in some anthocyanins compared to others [49]. Changes in environment, weather patterns, and soil characteristics could have a role in this observed difference [33]. In red rice, low amounts of anthocyanins are detected, between 0 to 93.5 mg anthocyanins/kg dry kernel [28,50,53,55]. The colour of red rice, however, is not caused by elevated levels of anthocyanins in the kernel, but is rather due to higher levels of proanthocyanidins. Proanthocyanidins are conjugated tannin structures [56]. Chen [53] was able to identify malvidin-3-glucoside in red rice extracts. However, other studies had no conclusive results as to the anthocyanin profile, indicating that if any anthocyanins are present, they exist in low levels. Conversely, purple rice has significantly higher levels of anthocyanins than what was found in red rice, with an average between 68 to 4700 mg anthocyanins/kg dry kernel [49,56]. With HPLC-PDA, cyanidin-3-glucoside, peonidin-3-glucoside, cyanidin-3-galactoside, and cyanidin-3-rutinoside were identified, as well as other assorted cyanidin and peonidin derivatives [49,56]. Finally, black rice has the highest recorded TAC of all the coloured rice samples studied, with an average between 79.5 to 5101 mg anthocyanins/kg dry kernel weight [28,50,52,53,54,55]. The main anthocyanins in black rice are cyanidin-3-glucoside and peonidin-3-glucoside. These two anthocyanins were identified and quantified through HPLC methods [49,50,53,56]. Other studies [50,56] have also found the presence of malvidin and cyanidin-3-rutinoside. With HPLC-PDA-MS, a greater diversity of anthocyanins was identified. The detected anthocyanins included (in order of prevalence) cyanidin-3,5-diglucoside, cyanidin-3-glucoside, cyanidin-3-(6″-*p*-coumaryl) glucoside, pelargonidin-3-glucoside, peonidin-3-glucoside, peonidin-3-(6″-*p*-coumaryl) glucoside, and cyanidin-3-arabidoside [52]. However, inter-subspecies variation in anthocyanin profile exists. When comparing black rice japonica and indica subspecies, for example, the cyanidin-3-glucoside:cyanidin-3-rutinoside:peonidin-3-glucoside ratio changed depending on subspecies as well as rice type (glutinous vs. non-glutinous) [54]. The average cyanidin-3-glucoside content of japonica subspecies was found to be 45% higher than the levels detected in indica subspecies, and 37% higher in non-glutinous black rice types than in the glutinous varieties [54].

## 4. Processing of Coloured Cereals

Rather than focusing on refined white flours to produce cereal food products, whole grain ingredients are needed to benefit from the potential health effects of anthocyanin-rich cereals as these molecules are predominantly found in the outer kernel layers that are typically removed to produce refined white flours. A deeper study of food products made with whole grain ingredients from coloured cereals is needed to gain insight into the fate of anthocyanins during cereal processing. It is well understood that anthocyanins are sensitive to environmental stresses, including exposure to heat, UV light, pH changes, and high oxygen levels, among other factors [9]. The processing of coloured cereals could therefore result in drastically reduced anthocyanin quantities. While further research is certainly necessary to better understand the effects of processing, some work has already begun in this field. The incorporation of bran has implications on the workability and acceptability of processed cereal products, and this must be considered when developing products which include these materials [27,57].

Bread was baked using purple and blue wheat grains, at two different time–temperature combinations [58]. A low temperature, long time baking profile (180 °C, 31 min) had a more significant (negative) effect on anthocyanin content than a high temperature, short time baking profile (240 °C, 21 min) [58]. Significant differences were also identified in the TAC between the wholemeal starting material and the final baked breads, quantified as a 7.1 and 72.8% TAC decrease for blue and purple wheat, respectively [58]. The anthocyanins of the purple varieties were shown to be more sensitive to degradation than the anthocyanins in the blue varieties [58]. In bread fortified with extracted black rice anthocyanins, the presence of the extract compounds was shown to have some effect on in vitro digestion time, with slower digestion reported in a mechanical digestion system for breads fortified with the anthocyanin extracts [59]. Slower digestion times allow for increased satiety [59]. Purple wheat bran has also been utilised to produce muffins [60]. No anthocyanins were detected in the muffins after baking [61]. This result was theorised to be due to the thermal treatment of baking, but could have also been a result of the dilution of total anthocyanins by other ingredients in the muffins, or the difficulties encountered when extracting water-extractable compounds from a high-lipid matrix [61]. Purple durum wheat was utilised in commercial fresh and dried pasta production to understand the effects of commercial pasta production processes on the anthocyanin content [57]. The results showed that milling, drying, and pasteurisation processes all affected the TAC of the pasta samples [57]. In particular, the drying process significantly reduced the TAC of the pasta with respect to the pasteurisation process for fresh pasta production [57]. In both cases, a decrease in the in vitro glycaemic index was found [57]. No mention of the TAC was made after cooking [57]. Purple wheat grains have also been used to produce alcoholic beverages. Beer and liqueur were produced using “antho-grain” purple wheat bran, and the TAC and antioxidant capacity of the beverages were tested [62,63]. In both beverages, antioxidant activity, total phenolic content, and TAC were found to increase due to the use of purple grains as the fermentation substrate [62,63].

Blue maize was utilised to produce cookies [64]. The effects of added acidulants and cooking time–temperature relationships on TAC were examined [64]. It was found that the addition of citric acid resulted in an increased preservation of anthocyanins in the cookies [64]. The addition of acidulants to maintain a low pH increased the stability of the anthocyanins, maintaining a pink colour and reducing colour loss [64]. In addition, the results agreed with earlier works, which found that high temperature, short time thermal processing was preferable for the preservation of anthocyanins in a complex food matrix [58,64]. The effect of extrusion on the TAC of whole grain blue maize extruded snacks has also been studied [65]. It was found that during the process, although several chemical modifications occur to anthocyanins (including degradation), relatively high anthocyanin retention values were found for extruded snacks [65].

The effect of cooking and storage conditions on coloured rice has also been examined. While cooking diminished the overall colour of the rice kernels, the solvent extractable anthocyanin content was found to increase after cooking [66]. This indicates that cooking might enhance the destruction of cell wall structures in the hull layer, further increasing extractability, which may overcome the negative effects of thermal treatments on the TAC in the extracts [66]. The impact of storage conditions on cooked and raw grains, conversely, was found to be negligible [66].

## 5. Antioxidant Potential and Health Effects

Free radicals and other reactive species produced in the body under physiological conditions play important roles in regulatory pathways and processes [67]. Production of free radicals and other reactive species is generally regulated by cellular defence systems, at a controlled rate. However, if the antioxidant defence system is disturbed and is not working sufficiently, an imbalance occurs in cells towards oxidative stress [2,67]. This state has been linked to the development of a number of diseases including cancers, cardiovascular diseases, hypertension, inflammation, and diabetes [3,67,68]. Dietary antioxidants are able to mitigate this oxidative stress through a number of mechanisms, depending on the structure of the molecule. Antioxidants may be efficient free radical scavengers, singlet oxygen quenchers, peroxide inactivators, metal ion chelators, and/or inhibitors of pro-oxidative enzymes [3,67,68]. Many phytochemicals in our diet act as antioxidants. Anthocyanins are one of these phytochemicals. Indeed, anthocyanins have even demonstrated a greater antioxidant activity than vitamins C and E in vitro [42]. However, the exact mechanism that governs their antioxidant effect is still under debate and requires further study [69]. Despite this mechanistic uncertainty, numerous in vivo and in vitro studies have demonstrated an antioxidative effect of anthocyanins [68,69].

### 5.1. Antioxidant Potential of Anthocyanins

Consumption of whole grain cereals has been shown to protect against obesity, diabetes, cardiovascular disease, and cancer [1,3]. Part of these health benefits can be ascribed to the high dietary fibre content. Phenolic compounds in general have also demonstrated positive effects on these health outcomes [2,3]. Due to the complex nature of these effects, and although attempts have been made to investigate the specific role of individual compounds, the specific health-promoting effect of each component has not yet been fully elucidated [2,3]. The question, hence, remains as to whether each compound exerts its effect individually, or synergistically with other compounds, such as dietary fibre and specific food structures [2,3]. The synergistic effect of anthocyanins and other compounds (e.g., the total phenolic content of cereals) on the antioxidant potential of cereals has been explored in a number of studies [6,12,39,53,54,55,70,71,72]. In addition, often, more than one antioxidant capacity index is measured or reported for the same food/bioactive compound to capture different underlying mechanisms for free radical scavenging capacity [39]. This, however, has caused difficulties in the interpretation of data [39]. The ORAC assay, due to its biological relevance, is a widely used method to investigate antioxidant capacity against peroxyl radicals [24]. DPPH radical assays are also widely used, with the focus of this test being on hydrogen-donating antioxidants against nitrogen radicals [24]. In addition, other assays such as TEAC and FRAP are sometimes utilised, further complicating analysis and data comparison.

Consensus has not yet been reached on the significance of anthocyanins in the overall antioxidant potential of whole grain cereals. Bellido [39] found that the antioxidant potential of extracts from barley was highly correlated with the TAC detected in the kernel. In another study that focused on the antioxidant activity of anthocyanin standard solutions, anthocyanin pigments possessed antioxidant capacities (in order of power: peonidin-3-glucoside, malvidin, cyanidin-3-glucoside, petunidin-3-glucoside) [53]. This study, however, also reported that the antioxidant capacity of anthocyanins was much weaker than the antioxidant capacity measured for other phenolic compounds. In whole black rice grains, it was shown that the TAC contributed only 0.5 to 2.5% of the total antioxidant potential of the grains (Table 4) [53]. These results were contradicted in a later study where purple rice bran was shown to have the highest ORAC values, and that this correlated to the high total flavonoid and total phenolic contents, relative to other coloured grains (Table 4) [49]. Zilic [47] found similar results in maize kernels, and attributed the effects to an overall higher phenolic content due to the presence of anthocyanins. A general relationship between the enrichment of anthocyanins and the overall antioxidant potential has been observed in other reports as well, with correlations being established between the TAC, total phenolic content, and the antioxidant potential [40,45,46,48]. As can be seen in Table 1 and Table 2, the full dataset on the TPC (Total Phenolic Content), antioxidant potential, and TAC can often not be found in research papers on coloured wheat and barley varieties. Conversely, the full dataset is often provided for maize and rice varieties (Table 3 and Table 4). These data can be used to identify correlations between TAC, antioxidant potential, and total phenolic content. The lack of correlation observed in some studies may be due to the types of materials (e.g., whole kernel, flour, bran, etc.) used, assessment methods for antioxidative capacity, anthocyanin extraction methods, and the utilisation of complex extracts with more than one bioactive [48]. The variations in results may also be due to the complex nature of the antioxidant mechanisms. Anthocyanin compounds may act more significantly in synergy with other compounds to promote the overall antioxidative capacity, rather than working as independent antioxidants. Additionally, the anthocyanin profiles of different cereal grains, and even different varieties of the same grain, are widely variable. This should be acknowledged when comparing antioxidant capacities, due to the potential relationship between structural variations in anthocyanins (e.g., acylation, glycosylation, etc.) and their antioxidant capacity. More research is needed on the antioxidant potential of anthocyanins in cereals, especially wheat, in order to further understand these relationships and assess the potential of these coloured cereals to produce healthy food products.

### 5.2. Human and Animal Studies on the Health Effects of Cereal-Based Anthocyanins

In a review of health benefits associated with anthocyanin consumption, evidence was provided for the beneficial effects of anthocyanins and their metabolites [2,68,69]. Protection of the vascular endothelium can directly and indirectly limit long-term stroke effects, and significantly lower blood pressure [68,69]. Recently, the role of anthocyanin intake on the modulation of gut health—through and independently from the gut microbiome—has been elucidated [68,69]. Additionally, emerging evidence connects anthocyanin consumption to bone health [2,69]. Cereal-based anthocyanins have also been the subject of both in vivo and in vitro research [73,74,75,76,77,78,79,80,81,82]. However, to date, the mechanism of the health benefits they confer is unknown. While the antioxidant potential of anthocyanins could be responsible for positive health outcomes, as stated above, other theories have suggested that it is rather digestive breakdown products (such as phenolic acids) that provide the observed effects [75]. Moreover, the necessary dosage, source, and format of supplementation must be further researched and standardised to obtain enough data to formulate accurate advice on the consumption of cereal-based anthocyanins [68]. Limitations in trials to date include large variability in the administered dose, as well as short trial durations [68]. Longer duration trials to assess dose–response relations are needed to adequately determine whether a supplementation effect exists, although no adverse effects have been reported [68]. It should also be taken into account that, in general, many phenolic compounds are present simultaneously in foods of plant origin, and it can be difficult to establish which compound specifically exerted the observed effects [75]. It is therefore a necessity to identify reliable and efficient biomarkers for the ingestion of anthocyanins to demonstrate bioefficacy [75]. Current research has centered on the addition of purified extracts to the diet of animal models.

Human intervention studies and animal models using berries, parts of plants, coloured cereals, and vegetables, or purified anthocyanin-rich extracts from these sources, have demonstrated significant improvements in low-density lipoprotein (LDL) oxidation, very low-density lipoprotein (VLDL), C-reactive protein (CRP), total triglycerides, and other biomarkers of CVD (Cardiovascular Disease) in the blood, as well as decreasing comorbidities—all of which aid in the amelioration of CVD [68,75,82]. Some clinical trials have demonstrated improved clinical states in patients who receive polyphenol supplementation (varying amounts) with CVD, when polyphenol supplementation occurs [82]. Supplementation of black rice pigment fractions to the diet significantly inhibited atherosclerotic plaque formation in rabbits and apolipoprotein E-deficient mice, and it was found that these properties were not derived from the dietary fibre and vitamin E components of the rice bran [80,82,83,84]. In further studies, purified anthocyanin extracts were utilised to confirm the role of anthocyanins in the observed anti-atherosclerotic effect [80]. Isolated cyanidin-3-glucoside and peonidin-3-glucoside showed antioxidative and anti-inflammatory activities in vitro [85]. Dietary supplementation of anthocyanin-rich extracts from black rice for apolipoprotein E-deficient mice resulted in a significant atheroprotective ability—both by inhibiting plaque progression, and increasing the stability of vulnerable plaque [80]. In addition, supplementation caused a 60% decrease in serum triglyceride content and non-high-density lipoprotein cholesterol—important markers in heart disease, since increased lipid levels are considered to be important risk factors for the development and progression of plaque [80]. It is theorised that the improvement in plaque stability and inflammatory responses was due to an improved lipid profile [80]. Anthocyanin-rich extract from black rice was also shown to have a positive effect on hyperlipidaemia in fructose-fed mice, based on a number of biomarkers [77]. In mice fed a high-fat diet, black rice anthocyanin extract (when consumed daily for 12 weeks), was shown to alleviate hypercholesterolaemia [76]. Anthocyanin-rich purple corn extracts were also found to suppress the mRNA levels of enzymes involved in free fatty acid and triglyceride synthesis [77].

In addition, cereal anthocyanins have been found to prevent high-fat diet-induced alterations in animal models. Mice fed a high-fat diet supplemented with isocaloric white, purple, or black whole wheat for 12 weeks were analysed for a number of biomarkers [79]. Black wheat supplementation significantly reduced body weight gain and fat pad, while both black and purple wheats reduced total cholesterol, serum triglyceride, and serum free fatty acid levels, while restoring normal blood glucose and insulin resistance levels [79]. In addition, the enzyme expression of fatty acid balancing, ß-oxidation, and oxidative stress response enzymes was significantly elevated with the consumption of coloured wheats, which led to positive biochemical and physiological outcomes [79]. The analysis of adipose and liver tissues also revealed the activation of multiple pathways and genes related to fatty acid-ß oxidation, antioxidative enzymes, and the balancing of fatty acid metabolism in black wheat-supplemented mice [79]. These results together indicate that the incorporation of black wheat in the diet can prevent obesity and related metabolic outcomes [79]. In vitro supplementation of cyanidin-3-glucoside on cell cultures was shown to ameliorate insulin resistance-related endothelial dysfunction caused by lipotoxicity [86]. The supplementation of anthocyanin-rich extract from black rice additionally increased insulin sensitivity in fructose-fed mice, supporting this conclusion [77]. Again, while the underlying mechanism may be mainly related to the inhibition of oxidative stress and improvement of the plasma lipid profile, the results indicate that supplementation with cereal-based anthocyanins may possess clinical importance in preventing the metabolic syndrome by ameliorating the negative effects of high-fat or high-sugar diets, which lead to this negative health outcome [77,79]. Furthermore, along with the prevention of chronic non-communicable diseases such as obesity, diabetes, and cardiovascular disease, anthocyanin supplementation can mitigate the symptoms of these diseases. Diabetes often causes chronic inflammation, hypertrophy, apoptosis, and fibrosis in the heart, subsequently leading to deterioration in cardiac function [74]. Previous research has shown anthocyanins possessing cardioprotective properties [74,75,80,83,84]. In rats with streptozotocin-induced diabetes, treatment with anthocyanin-rich purple rice extract (73% cyanidin-3-glucoside) showed a significant reduction in cardiac hypertrophy and fibrosis; therefore restoring the deteriorating cardiac functions in diabetic rats, as determined from heart functional parameters [74]. In vivo, anthocyanin content was positively correlated with pancreatic lipase inhibitory activity [73].

Along with the prevention and treatment of chronic non-communicable diseases, cereal-based anthocyanins have shown promising effects on other health outcomes. Anthocyanin-rich extract from black rice improved the relative abundance of gut microbiota in mice with a high cholesterol diet. However, the extract was unable to restore the relative levels of *Lactobacillus* and *Bifidobacterium* in antibiotic-treated mice. Therefore, the gut modulatory effects of anthocyanins are dependent on the presence of a healthy gut microbiota [76]. Crude anthocyanin extracts from black rice were found to promote CD3, CD19, CD11-b, and Mac-3 cells in leukaemic mice, as well as promote macrophage phagocytosis, and decrease NK cell activity at doses above 20 mg/kg [87]. Purified anthocyanin extract from purple highland barley exhibited high antioxidative activity and potential neuroprotective effects on induced hypoxic damage in vitro [88]. Anthocyanin extract maintained cell viability, restored cell morphology, inhibited lactic dehydrogenase leakage, and reduced reactive oxygen species, among other effects [88]. Cells treated with anthocyanin extract were found to activate autophagy, indicating that it is a potential survival mechanism against hypoxia-induced injury, and therefore could be a preventative agent for brain dysfunction caused by hypoxic damage [88]. Finally, anthocyanins have also been investigated as a protective agent against aging and aging-affiliated pathology [78,81]. In nematodes, it was found that supplementation with anthocyanin-rich extracts of purple wheat extended the mean lifespan of wild and mutant species by 10% and 9%, respectively [78]. Lifespan extension was found to depend on the DAF-16 transcription factor [78]. Purple wheat increased stress response and reduced oxidative stress [78]. Additionally, black rice anthocyanins were found to minimise senescence in mice by altering endogenous antioxidant enzymatic and aging-related enzymatic activities, and regulating superoxide dismutase 1,2 and MAO-B gene expressions [81].

## 6. Conclusions

Certain cereals, in particular wheat, barley, maize, and rice, have coloured varieties that contain substantial levels of anthocyanins in the outer kernel layers. Utilisation of whole grain ingredients derived from these coloured cereals for the production of functional foods is, hence, a strategy worth exploring. In this review paper, research reporting on the content and types of anthocyanins detected in coloured cereals was summarised. In addition, the potential health benefits of anthocyanins and their antioxidant potential were discussed. To date, there is only limited evidence to show that the processing of coloured whole grain cereal ingredients into anthocyanin-containing food products is possible. More studies are needed to gain insight into the fate of anthocyanins enclosed in cereal structures during food processing in order to develop processing strategies that would better retain these molecules in final products reaching the consumer. In addition, besides developing such strategies, it remains to be studied if these cereal anthocyanins would also offer health benefits to consumers beyond those of consuming current whole grain products. There is some evidence from in vitro and animal and human studies to support the beneficial effect of cereal-based anthocyanins on a variety of health outcomes such as obesity, diabetes, aging, cancer, and cardiovascular disease. However, more research is necessary to determine the true effects of anthocyanins in humans. Additionally, most studies utilised purified extracts to test health effects. However, this is an unrealistic medium for the consumption of cereal-based anthocyanins. More research is needed to elucidate the effect of consuming anthocyanins within a processed cereal matrix.

## Figures and Tables

**Table 1:** Selection of studies focusing on total anthocyanin content (TAC) in various wheat cultivars. The anthocyanin quantification method and assay used for antioxidant potential are also listed.

Wheat Colour	Wheat Variety	TAC (mg/kg Kernel Weight)	TAC Measurement	Antioxidant Potential	Total Phenolic Content
Purple	Laval [28]	96 ± 5	Spectrophotometer	n.d.	n.d.
Purple	Laval 19 [31]	10	pH Differential	n.d.	n.d.
Purple	Charcoal [31]	305	pH Differential	n.d.	n.d.
Purple	Indigo [24]	72 ± 1	Spectrophotometer	3959 µmol TE/100 g *(ORAC)*	160 µmol FAE/100 g
Blue	Purendo [37]	212 ± 3	Spectrophotometer	n.d.	n.d.
Blue	Purendo [28]	18 ± 4	Spectrophotometer	n.d.	n.d
Blue	BVAL-214025 [38]	74 ± 2	Spectrophotometer	5 mmol Fe2 + equiv/100 g *(FRAP)*	163 mg FAE/100 g
Black	BW/2 PBW621-F5 [30]	198 ± 9	Spectrophotometer	n.d.	n.d.
Black	Black Wheat [30]	186 ± 17	Spectrophotometer	n.d.	n.d.
Black	UC 66049/Konini [31]	56	pH Differential	n.d.	n.d.

**Table 2:** Selection of studies focusing on total anthocyanin content (TAC) in various barley cultivars. The anthocyanin quantification method and assay used for antioxidant potential are also listed.

Barley Colour	Barley Variety	TAC (mg/kg Kernel Weight)	TAC Measurement	Antioxidant Potential	Total Phenolic Content
Purple	CI-1248 [39]	573 ± 26	HPLC	3937 µmol TE/100 g *(ORAC)*	n.d.
Purple	Yu 5904-088 [40]	679 ± 19	Spectrophotometer	78% *(DPPH)*	n.d.
Blue	Kompolti korai 1 [41]	84 ± 3	Spectrophotometer	n.d.	n.d.
Blue	Ubamer [40]	77 ± 2	Spectrophotometer	35% *(DPPH)*	n.d.
Blue	Tankard [28]	345 ± 1	Spectrophotometer	n.d.	n.d.
Black	Peru-35 [39]	0	HPLC	5430 µmol TE/100 g *(ORAC)*	n.d.
Black	Black [40]	8 ± 1	Spectrophotometer	29% *(DPPH)*	n.d.

HPLC: High-performance liquid chromatography.

**Table 3:** Selection of studies focusing on total anthocyanin content (TAC) in various maize cultivars. The anthocyanin quantification method and assay used for antioxidant potential are also listed.

Maize Colour	Maize Variety	TAC (mg/kg Kernel Weight)	TAC Measurement	Antioxidant Potential	Total Phenolic Content
Purple	KKU-WX211003 [45]	89 ± 1	pH Differential	14 μmol TE/g DW *(DPPH)*	7 mg GAE/g DW
Purple	AREQ516540TL [46]	850 ± 6	Spectrophotometer	92% *(ABTS)*	3400 mg GAE/100g
Purple-black	KKU-WX111031 [45]	1439 ± 8	pH Differential	22 μmol TE/g DW *(DPPH)*	20 mg GAE/g DW
Purple-yellow	KKU-WX211004 [45]	27 ± 1	pH Differential	12 μmol TE/g DW *(DPPH)*	6 mg GAE/g DW
Red-yellow	ZPL-5 [47]	3 ± 0	HPLC	23 mmol TE/kg *(TEAC)*	6011 GAE/kg DM
Dark red	ZPP-1 selfed [47]	696 ± 3	HPLC	27 mmol TE/kg *(TEAC)*	6115 GAE/kg DM
Light blue	ZPP-2 selfed [47]	379 ± 5	HPLC	36 mmol TE/kg *(TEAC)*	10529 GAE/kg DM
Blue	Blue [46]	100 ± 2	Spectrophotometer	63% *(ABTS)*	343 mg GAE/100 g
Black	Black [46]	77 ± 2	Spectrophotometer	60% *(ABTS)*	457 mg GAE/100 g
Black	Negro normal OLI 04 PV [46]	120 ± 2	Spectrophotometer	85% *(ABTS)*	544 mg GAE/100 g

GAE: Gallic acid equivalents; DW: dry weight; DM: dry matter.

**Table 4:** Selection of studies focusing on total anthocyanin content (TAC) in various rice cultivars. The anthocyanin quantification method and assay used for antioxidant potential are also listed.

Rice Colour	Rice Variety	TAC (mg/kg Kernel Weight)	TAC Measurement	Antioxidant Potential	Total Phenolic Content
Purple	GPNO 20,175 [49]	68 ± 29	Spectrophotometer	98 µmol TE/g bran *(ORAC)*	6 mg GAE/g bran
Purple	Hung Hsien Ju PI 16,097 [49]	151 ± 25	Spectrophotometer	174 µmol TE/g bran *(ORAC)*	12 mg GAE/g bran
Purple	Hung Tsan [49]	199 ± 36	Spectrophotometer	147 µmol TE/g bran *(ORAC)*	11 mg GAE/g bran
Purple	IAC600 [51]	4700	Spectrophotometer	101 µmol TE/g *(ORAC)*	6 mg GAE/g
Red	SB7 [50]	0	pH Differential	75 µmol TE/g *(ORAC)*	1 mg GAE/g
Red	Red [28]	94 ± 1	Spectrophotometer	n.d.	n.d.
Black	Asamurasaki [52]	1400 ± 41	HPLC	n.d.	n.d.
Black	Asamurasaki [53]	474	HPLC	65 mmol TE/g DW *(ORAC)*	n.d.
Black	Okunomurasaki [53]	80	HPLC	55 mmol TE/g DW *(ORAC)*	n.d.
Black	Longjin 01 [54]	5101 ± 79	pH Differential	1138 mmol TE/g DW *(ORAC)*	4766 mg GAE/100 g DW
